# Postcoital visual loss due to valsalva retinopathy: A case report

**DOI:** 10.1016/j.amsu.2022.104721

**Published:** 2022-09-21

**Authors:** Ahmed Mahjoub, Nadia Ben Abdesslem, Atf Ben Abderrazek, Anis Mahjoub, Dorra Ben Ammar, Chirine Bachraoui, Abdelkarim Bouatay, Emna Bouslama, Mohamed Ghorbel, Hachemi Mahjoub

**Affiliations:** a Department of Ophthalmology, Farhat Hached Hospital, University of Sousse, Tunisia; b Department of Clinical Hematology, Farhat Hached Hospital, University of Sousse, Tunisia

**Keywords:** Case report, Valsalva retinopathy, Sexual activity, Nd:YAG laser hyaloidotomy, Swept source OCT, Nd:YAG, Neodymium:yttrium-aluminum-garnet, OCT, Optical coherence tomography

## Abstract

Valsalva retinopathy is a rare pathology presenting as a sudden and painless loss of vision affecting young subjects with no medical history. It is the result of an increase of intraocular venous pressure, leading to retrohyaloid haemorrhage.

We describe here the clinical presentation of a retrohyaloid hemorrhage resulting from a valsalva mechanism following a sexual activity, in a 28-year-old patient treated by Neodymium:yttrium-aluminum-garnet (Nd:YAG) laser hyaloidotomy with a visual recovery of 20/20 on 3 weeks follow-up.

Valsalva retinopathy is a rare and an easy-to-diagnose pathology that is safely handled by Nd:YAG laser hyaloidotomy for a quick visual acuity recovery.

## Highlights

• Report a case of a valsalva retinopathy occuring in a young healthy adult following a sexual activity.
• Develop our personal experience and how we managed to jugulate the hemorrhage by a Nd-yag laser membranotomy.
• Describe the mechanisms leading to Valsalva retinopathy and its management by a Nd-yag laser membranotomy.

## 1 Introduction and importance

The Valsalva maneuver is the forced exhalation against a closed glottis, leading to increased intra-thoracic and intra-abdominal pressure and raised central venous pressure. Valsalva retinopathy is a rare pathology presenting as a sudden and painless loss of vision affecting young subjects with no medical history. It is the result of an increase in intraocular venous pressure, leading to intravitreal, or more frequently, retrohyaloid hemorrhage [1].

We describe here the clinical presentation of a retrohyaloid hemorrhage resulting from a valsalva mechanism following sexual activity, in a 28-year-old patient and present the modalities of its management.

This case report has been reported in line with the SCARE criteria [2].

## 2 Case presentation

We report here the case of 28-year-old immunocompetent caucasian patient, with no significant medical history, who presented at our department of ophthalmology in Sousse Tunisia, with sudden and isolated loss of vision in the right eye. There was no history of trauma, redness, flashes, floaters, or transient visual loss in the past.

**Fig. 1:**
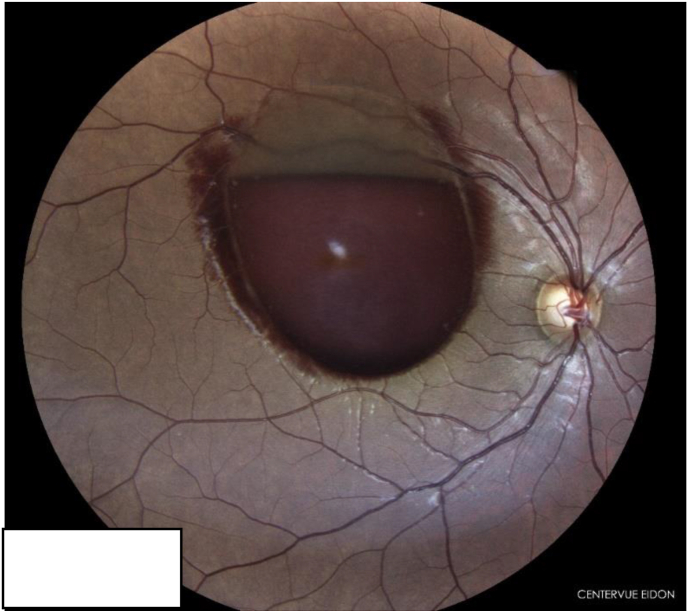
Color photo fundus demonstrates a large subhyaloid hemorrhage covering the macular region with a horizontal upper level and an arciform lower limit. (For interpretation of the references to colour in this figure legend, the reader is referred to the Web version of this article.)

Ocular examination exhibited a visual acuity limited to counting fingers in the right eye and 20/20 in the left eye. Anterior segment examination was normal in both eyes. Dilated fundoscopy of the right eye showed a large circumscribed subhyaloid hemorrhage covering the macular region with a horizontal upper level and an arciform lower limit (Fig. 1).

**Fig. 2:**
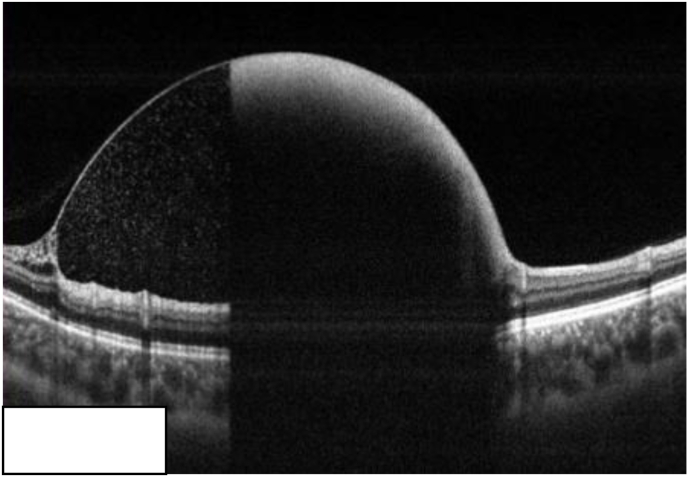
Swept source OCT shows shadow effect of the preretinal hemorrhage covering the macula, obstructing the image of the underlying retina and subretinal space.

Swept source OCT revealed shadow effect of the preretinal hemorrhage covering the macula, obstructing the image of the underlying retina and subretinal space (Fig. 2).

All investigations including complete blood counts, clotting parameters, biochemical parameters such as urea, creatinine and glucose were normal. Systemic evaluation was carried out; no abnormality was detected.

After further questioning about any recent strenuous activities, the patient admitted developing the vision loss immediately after sexual intercourse. With this history and examination findings, the diagnosis of Valsalva retinopathy following sexual activity was made.

On the following day, Nd:YAG membranotomy was applied in single shots resulting to an efficient photodisruption. We noted that the majority of blood drained into the inferior vitreous (Fig. 2 A).

Three hours later, fundoscopy showed inferior vitreous hemorrhage with total clearing of the macular region (Fig. 2B). The patient was then discharged and no medication was given.

**Fig. 3:**
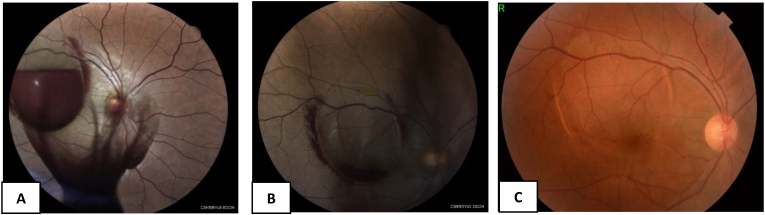
A: Color photo fundus 30 seconds following Nd YAG hyalodotomy, the hemorrhage drains inferiorly into vitreous, B: Color photo fundus 3 hours following Nd YAG hyalodotomy shows the inferior vitreous hemorrhage with complete clearing of the macular region, C: Color photo fundus 3 weeks following Nd YAG hyalodotomy shows complete clearing of the hemorrhage.. (For interpretation of the references to colour in this figure legend, the reader is referred to the Web version of this article.)

Three weeks after the procedure, visual acuity improved to 20/20 (Fig. 3). Dilated fundus examination showed complete clearing of the hemorrhage. The retina remained flat without the occurring of any complications.

This case report has been reported in line with the SCARE criteria [2].

## 3 Clinical discussion

Valsalva retinopathy is an uncommon disease, caused by a Valsalva mechanism, leading to high intraocular venous pressure causing intraocular bleeding. It frequently appears after a physical effort with a closed glottis [3].

The resulting sudden increase in intra-thoracic and abdominal pressure is transmitted through the downstream cephalic venous systems to the intraocular veins resulting in spontaneous rupture of the perifoveolar capillaries and formation of a preretinal hematoma [4,5].

It generally occurs during an effort of coughing, vomiting, carrying a heavy load, constipation, birth labor [6], and medical acts such as general anesthesia [7]. Most of the time, it is a single and, usually unilateral damage [8], typically seen in young males [9].

Sexual activity is known to be a risk factor for Valsalva retinopathy.

Sexual intercourse can be the source of various hormonal, hematological, and mechanical changements on the hemodynamic system of the organism, which can be a risk factor for valsalva retinopathy [[10], [11], [12], [13], [14]].

The association between sexual activity and retinal hemorrhage was first described by Friberg et al., in 1995 [13]. In 2009, Mahdi et al. described a serie of 10 patients who developed premacular hemorrhage resulting from vigorous sexual activity among 21 patients. Only one patient was managed with vitrectomy. Nd:YAG laser hyaloidotomy was performed in 8 patients [14].

The diagnosis of valsalva retinopathy is guided by the clinical presentation in the fundus examination of a solitary, well-circumscribed, unilateral hemorrhage in the macular region of an apparently healthy, associated with a history of Valsalva stress [15].

Hemorrhage can occur in any layer: subretinal, intraretinal, or preretinal: sub-internal limiting membrane [ILM], subhyaloid, or into the vitreous. OCT imaging is useful in the structural localisation of the hemorrhage, in management and follow-up [16].

There are three attitudes to consider when faced with a macular preretinal hemorrhage: monitoring, Nd:YAG laser posterior hyaloidotomy, or vitrectomy.

While conservative treatment is common for smaller hemorrhages, intervention must be considered if the hemorrhage is near the macula, persistent and located in the subhyaloid or subILM space [[17], [18], [19], [20]].

Preretinal hemorrhages secondary to Valsalva retinopathy usually resolve spontaneously in a few weeks to several months.

If monitoring is chosen as conservative treatment, the patient should avoid strenuous activities to prevent recidive, and sleep in sitting position to promote blood settling. However, the young age of most patients requires a rapid visual recovery in order to allow a quick rehabilitation [19].

Although the hemorrhage may regress spontaneously, it exposes to a risk of secondary preretinal fibrosis [21]. A slowly resolving subhyaloid hemorrhage also prolongs the contact of the retina with hemoglobin and iron, potentially leading to toxic damage to the retina, which can cause permanent visual loss [22].

Authors usually recommend a maximum delay of 3 weeks, before the organization of the collection makes its intravitreal evacuation impossible [23].

In this case, our patient received his procedure a day after the onset of his symptoms and was, therefore, treated within previously published time frames where normal visual acuity could be expected.

For safety reasons, it is essential in YAG laser treatment, to select lesions with a size superior to 3 Disk Diameter (DD), to minimize the risk of macular complications induced by the mechanical trauma of the photo-disruptive laser [19].

The power of the laser impacts should be gradually increased until a successful hemorrage drainage into the vitreous cavity is achieved, without exceeding 9 Mj. The impacts should be performed far from large blood vessels and from the fovea, at the inferior margin of the hemorrhage [24].

In deed, high laser power increases the risk of vision threatening retinal damage such as macular hole, macular tear and, retinal detachment [26].

To avoid such complications, careful patient selection should be performed: hemorrhages not larger than 3 disk diametres, precise laser focussing on the surface of the hemorrhage, and laser power not excessing 9 mJ [24,25].

The maximum power required in this case to perforate the posterior hyaloid membrane was 3.7 mJ and no retinal or choroidal damage was observed either immediately following the procedure or at follow-up.

It is admitted that the rapidity of recovery is correlated with the volume of the hematoma. In fact, most cases recover stable visual acuity by the end of the first month. Nevertheless, lesions exceeding 9DD can take three months to resorbate [17].

Ulbrig et al. conducted a retrospective review on 21 eyes with a premacular hemorrhage of various causes, treated with a pulsed Nd:YAG laser. In 16 eyes, visual acuity improved within 1 month. Four eyes required vitrectomy. Complications included a macular hole in one eye and a retinal detachment from a retinal break in a myopic patient and a single additional eye that developed a macular hole [26].

Rennie et al. showed a favorable outcome (VA > 6/12) in four on six treated patients and reported no ocular complications for 6 patients after laser membranotomy at 3–6 months follow-up [27].

Khan et al. reported a better visual outcome and no ocular complications for 11 patients after laser membranotomy at 6 month follow-up due to the absence of compromising factors such as diabetic retinopathy and vein occlusion [28].

Relative rest is recommended after the procedure to allow quick emptying of the hemorrhage into the vitreous cavity and thus reduce the risks of bleeding. However, after Nd:YAG laser hyaloidotomy, monitoring should be prolonged to 4 weeks to detect the eventual appearance of a secondary epiretinal membrane [29].

## 4 Conclusion

Sexual activity is a well known risk factor for Valsalva retinopathy.

Valsalva retinopathy remains a rare and easy-to-diagnose pathology that is safely handled by Nd:YAG laser hyaloidotomy for a quick visual acuity recovery.

## Ethical approval

We further confirm that any aspect of the work covered in this manuscript that has involved human patients has been conducted with the ethical approval of all relevant bodies and that such approvals are acknowledged within the manuscript. IRB approval was obtained (required for studies and series of 3 or more cases) Written consent to publish potentially identifying information, such as details or the case and photographs, was obtained from the patient(s) or their legal guardian(s).

## Source of funding

No funding was received for this work.

## Author contribution

Ahmed Mahjoub: writing the paper, Nadia Ben Abdesslem: data analysis, Atf Ben Abderrazek: writing the paper, Anis Mahjoub: collecting data, Dorra Ben Ammar: study concept. Chirine Bachraoui study design, Abdelkrim Bouatay: collecting data, Emna Bouslama: study design, Mohamed Ghorbel: correcting the final paper, Hachemi Mahjoub: correcting the final paper.

## Trail registry number

None.

## Guarantor

Atf Ben Abderrazek atf.benabderrazek@gmail.com.

## Availability of data and materials

All data and supplementary information are included in this published article.

## Abbreviations

Neodymium:yttrium-aluminum-garnet: Nd:YAG, SS OCT: Swept source optical tomography coherence.

## Authorship

All authors attest that they meet the current ICMJE criteria for Authorship.

## Patient consent

Written informed consent was obtained from the patient's legal guardian: The father, for publication of this case report and accompanying images. A copy of the written consent is available for review by the Editor-in-Chief of this journal on request”.

## Provenance and peer review

Not commissioned, externally peer reviewed.

## Declaration of competing interest

No conflict of interest exists. We wish to confirm that there are no known conflicts of interest associated with this publication and there has been no significant financial support for this work that could have influenced its outcome.
